# 新型冠状病毒感染疫情前后中国健康人群的巨细胞病毒、EB病毒感染情况分析

**DOI:** 10.3760/cma.j.cn121090-20240910-00342

**Published:** 2024-11

**Authors:** 芷凡 赵, 卓君 刘, 昱 王, 于谦 孙, 兰平 许, 晓辉 张, 晓军 黄, 旭颖 裴

**Affiliations:** 北京大学人民医院，北京大学血液病研究所，国家血液系统疾病临床医学研究中心，造血干细胞移植治疗血液病北京市重点实验室，北京 100044 Peking University People's Hospital, Peking University Institute of Hematology, National Clinical Research Center for Hematologic Disease, Beijing Key Laboratory of Hematopoietic Stem Cell Transplantation, Beijing 100044, China

**Keywords:** 巨细胞病毒, EB病毒, 新型冠状病毒, 感染, 抗体, Cytomegalovirus, Epstein-Barr virus, SARS-CoV-2, Infection, Antibodies

## Abstract

**目的:**

评估中国近10年健康人群巨细胞病毒（CMV）和EB病毒（EBV）感染情况，分析新型冠状病毒（以下简称新冠）感染疫情管控全面解除（以下简称解控）前后健康人群CMV和EBV感染情况的差异。

**方法:**

对2014年1月至2023年12月在北京大学人民医院进行造血干细胞移植前检查的8 827名健康移植供者的CMV和EBV的感染情况进行回顾性分析。运用Logistic回归分析CMV和EBV感染的危险因素。

**结果:**

8 827名健康移植供者中CMV和EBV的免疫球蛋白（Ig）G阳性率分别为94.52％和95.40％，显示出较高的既往感染率。CMV与EBV的既往感染率在解控前后差异无统计学意义（CMV：94.63％对93.92％，*P*＝0.250；EBV：95.36％对95.62％，*P*＝0.670），但既往感染人群IgG平均抗体滴度有所上升［CMV：（100.44±36.50）U/ml对（109.98±36.31）U/ml，*P*<0.001；EBV：（281.57±226.79）U/ml对（361.08±268.58）U/ml，*P*<0.001］。新冠疫情解控前后CMV IgM阳性率变化不显著，但EBV IgM阳性率升高（2.77％对6.29％，*P*<0.001），尤其解控3个月内EBV IgM阳性率达8.10％。进一步分析影响EBV IgM阳性率的因素，结果显示总体人群中性别（*OR*＝1.479，95％ *CI* 1.169～1.872，*P*＝0.001）、年龄［与<18岁组相比，18～50岁组（*OR*＝0.584，95％ *CI* 0.421～0.820，*P*＝0.002），>50岁组（*OR*＝0.389，95％ *CI* 0.248～0.610，*P*<0.001）］、新冠疫情解控（*OR*＝2.360，95％ *CI* 1.287～3.047，*P*<0.001）均是EBV IgM阳性的独立危险因素；按年龄段分组，<18岁组EBV IgM阳性率不受性别和新冠疫情解控影响，18～50岁组中性别（*OR*＝1.499，95％ *CI* 1.138～1.975，*P*＝0.004）和新冠疫情解控（*OR*＝2.608，95％ *CI* 1.940～3.507，*P*<0.001）均是EBV IgM阳性的独立危险因素，>50岁组中新冠疫情解控是EBV IgM阳性的独立危险因素（*OR*＝2.222，95％ *CI* 1.101～4.484，*P*＝0.026）。

**结论:**

中国健康人群CMV和EBV的既往感染率高，随着年龄增长感染率有所增加。新冠感染可能增加健康人群EBV激活率，其中18岁以上人群新冠感染对EBV激活率的影响更为显著。

## Abstract (en)

**Objective:**

This study aimed to assess the infection status of cytomegalovirus（CMV）and Epstein-Barr virus（EBV）in healthy populations in China over the past decade and analyze the differences in CMV and EBV infection and related risk factors in healthy populations before and after the lifting of coronavirus disease 2019（COVID-19）pandemic control measures.

**Methods:**

This study retrospectively analyzes the CMV and EBV infection status of 8 827 healthy donors who underwent pre-hematopoietic stem cell transplantation screening at Peking University People's Hospital from January 2014 to December 2023. Logistic regression analysis was conducted to determine the risk factors for CMV and EBV infection.

**Results:**

The CMV and EBV IgG positivity rates were 94.52％ and 95.40％ among the healthy donors, respectively, with no significant differences before and after the lifting of pandemic control measures（all *P* value>0.05）. However, IgG antibody titers increased［CMV:（100.44±36.50）U/ml *vs*（109.98±36.31）U/ml,*P*<0.001;EBV:（281.57±226.79）U/ml *vs*（361.08±268.58）U/ml, *P*<0.001］after lifting the COVID-19 restrictions. However, the CMV IgM positivity rate remained unchanged. The EBV IgM positivity rate significantly increased after lifting measures（2.77％ *vs* 6.29％, *P*<0.001）, reaching 8.10％ within 3 months. Further analysis of the factors affecting EBV IgM positivity revealed that gender（*OR*＝1.479, 95％ *CI* 1.169–1.872, *P*＝0.001）, age［compared with the group younger than 18 years, the 18–50-year age group（*OR*＝0.584, 95％ *CI* 0.421–0.820, *P*＝0.002）, the >50-year age group（*OR*＝0.389, 95％ *CI* 0.248–0.610, *P*<0.001）］, and the lifting of COVID-19 restrictions（*OR*＝2.360, 95％ *CI* 1.287–3.047, *P*<0.001）were independent factors influencing EBV IgM positivity in the general population. The EBV IgM positivity rate in individuals under 18 years old was not affected by gender or the lifting of COVID-19 restrictions when stratified by age group. Both genders（*OR*＝1.499，95％ *CI* 1.138–1.975, *P*＝0.004）and the lifting of COVID-19 restrictions（*OR*＝2.608, 95％ *CI* 1.940–3.507, *P*<0.001）were independent factors affecting EBV IgM positivity in the 18–50-year age group. The lifting of COVID-19 restrictions（*OR*＝2.222, 95％ *CI* 1.101–4.484, *P*＝0.026）was the sole independent factor affecting EBV IgM positivity in individuals over 50 years old.

**Conclusion:**

Previous infection rates of CMV and EBV are high in healthy populations in China, which increase with age. COVID-19 infection may increase EBV reactivation rates in healthy individuals, with a more pronounced effect on those aged >18 years.

人巨细胞病毒（CMV）和EB病毒（EBV）是疱疹病毒β、γ亚族的常见病毒，人群普遍易感[1]。大多数健康成年人感染CMV或EBV后无症状或仅有轻微症状，但对于免疫低下人群（如艾滋病患者、接受移植患者和新生儿），CMV、EBV感染可引起病毒血症甚至严重的终末器官疾病。尤其对于造血干细胞移植后早期患者，CMV和EBV感染率高，预后差[2]–[4]。移植供者的病毒血清学状态是影响移植后病毒再激活的重要因素，因此移植供者CMV和EBV的免疫球蛋白（Ig）G和IgM检测是移植前供者常规检查项目[5]–[7]。

既往研究表明，健康成人CMV既往感染率为60％～90％，EBV既往感染率超过90％[8]–[11]。但近10年我国缺乏大样本数据报道，且现有国内对于CMV、EBV的流行病学调查中多以抗体的定性结果，即病毒感染的阳性率作为研究结论或研究的影响因素，缺少对CMV、EBV抗体滴度的定量研究。

CMV和EBV的流行病学特征与年龄、性别、社会经济状况等多种人口因素有关[12]–[13]。近年来多篇文献[14]–[15]报道新型冠状病毒（以下简称新冠）感染会诱导EBV再激活，且新冠感染后引起的某些“长新冠”症状也认为可能与EBV的再激活有关。但目前关于新冠感染是否增加CMV和EBV激活缺乏大样本病例报道。

本文回顾性分析了我中心近10年健康移植供者CMV、EBV的IgG、IgM抗体阳性率和抗体滴度，同时以2022年12月为时间界线进一步分析了我国新冠感染疫情管控全面解除（以下简称解控）前后健康人群CMV和EBV感染情况差异和风险因素。

## 对象与方法

1. 供者资料：本研究收集2014年1月至2023年12月间在北京大学人民医院进行CMV和EBV的IgG和IgM检测的8 827名健康移植供者。男性5 830名，女性2 997名。以2022年12月为时间界线进一步分组，7 412名（83.97％）供者的EBV、CMV抗体在新冠疫情解控前检测，1 415名（16.03％）供者的EBV、CMV抗体在解控后检测（含2022年12月至2023年2月的284名）。

2. 检测方法和定义：采集外周血4 ml，采用化学发光免疫分析法检测血清中EBV壳体抗原（VCA）的特异性IgG、IgM抗体滴度以及CMV的特异性IgG、IgM抗体滴度。以CMV IgG≥12 U/ml、CMV IgM≥18 U/ml、EBV IgG≥20 U/ml、EBV IgM≥20 U/ml为阳性，其余滴度值视为阴性。当超出检测范围时，测定的抗体滴度以相应的最大值或最小值计算。CMV、EBV IgG阳性定义为既往感染，CMV、EBV IgM阳性定义为现症感染。

3. 统计学处理：使用SPSS 27.0及GraphPad prism 10软件进行数据分析，组间计数资料（性别、年龄构成、阳性率）比较采用*χ*^2^检验和Fisher精确检验，组间计量资料（年龄、抗体滴度）比较采用*t*检验。计量资料符合正态分布时以*x*±*s*表示，不符合正态分布时以*M*（*IQR*）表示。计数资料用构成比和百分率表示。用Logistic回归得到比值比（*OR*）估计值和相应的95％置信区间（*CI*）并进一步分析潜在的危险因素。双侧*P*<0.05为差异有统计学意义。

## 结果

1. 基本特征：见表1，8 827名健康移植供者中新冠疫情解控前7 412名，解控后1 415名。将入组人群按照年龄分组后，<18岁组751名，18～50岁组6 433名，>50岁组1 643名。人群的平均年龄为37.5岁。新冠疫情解控前后两组人群性别、年龄的差异均无统计学意义（均*P*>0.05）。

**表1:** 8 827名健康移植供者的基本特征

特征	总体（8 827名）	新冠解控前（7 412名）	新冠解控后（1 415名）	*P*值
性别（名，男/女）	5 830/2 997	4 906/2 506	924/491	0.517
年龄（岁，*x*±*s*）	37.5±13.4	37.4±13.4	37.9±13.5	0.171
年龄段分组（名）				0.086
<18岁	751	635	116	
18～50岁	6 433	5 427	1 006	
>50岁	1 643	1 350	293	

2. 健康移植供者总体CMV和EBV情况：CMV IgG和IgM的阳性率分别为94.52％、0.69％；阳性人群抗体滴度分别为（101.96±36.63）U/ml、（27.41±12.99）U/ml。其中CMV IgG的阳性率在<18岁组、18～50岁组、>50岁组中分别为80.69％、95.07％、98.66％（均*P*<0.001）；阳性人群抗体滴度分别为（83.55±32.67）U/ml、（100.78±36.19）U/ml、（113.30±36.17）U/ml（均*P*<0.001）；CMV IgM的阳性率在<18岁组、18～50岁组、>50岁组中分别为0.80％、0.65％、0.85％；阳性人群抗体滴度分别为（20.44±1.93）U/ml、（27.40±11.10）U/ml、（29.92±19.08）U/ml，阳性率与抗体滴度差异均无统计学意义（均*P*>0.05）。

EBV IgG和IgM的阳性率分别为95.40％、3.33％；阳性人群抗体滴度分别为（294.34±235.80）U/ml、（33.44±15.98）U/ml。其中EBV IgG的阳性率在<18岁组、18～50岁组、>50岁组中分别为86.28％、96.10％、96.84％（均*P*<0.001）；阳性人群抗体滴度分别为（254.07±212.11）U/ml、（286.04±231.81）U/ml、（343.02±252.87）U/ml（均*P*<0.001）。EBV IgM的阳性率在<18岁组、18～50岁组、>50岁组中分别为5.59％、3.34％、2.25％（均*P*<0.001）；阳性人群抗体滴度分别为（31.28±9.59）U/ml、（33.34±16.28）U/ml、（36.49±19.56）U/ml，差异均无统计学意义（均*P*>0.05）。

3. 新冠疫情解控前后健康移植供者CMV和EBV感染情况：CMV的IgG阳性率在新冠疫情解控前后分别为94.63％和93.92％（*P*＝0.250），阳性人群抗体滴度分别为（100.44±36.50）U/ml和（109.98±36.31）U/ml（*P*<0.001）；IgM阳性率分别为0.69％和0.71％（*P*＝0.940），阳性人群抗体滴度分别为（28.45±13.88）U/ml和（22.13±4.21）U/ml（*P*＝0.160）。

EBV的IgG阳性率在新冠疫情解控前后分别为95.36％和95.62％（*P*＝0.670）；阳性人群抗体滴度分别为（281.57±226.79）U/ml和（361.08±268.58）U/ml（*P*<0.001）；IgM阳性率分别为2.77％和6.29％（*P*<0.001）；阳性人群抗体滴度分别为（32.97±15.05）U/ml和（34.52±17.99）U/ml（*P*＝0.045）。新冠疫情解控3个月内284名健康供者EBV IgM阳性率达8.10％，阳性人群EBV IgG和IgM抗体滴度分别为（355.11±274.91）U/ml和（39.04±22.20）U/ml。

4. 新冠疫情解控前后不同年龄段健康供者CMV和EBV感染情况：如表2所示，CMV的IgG阳性率和IgM阳性率在新冠疫情解控前后在<18岁组、18～50岁组、>50岁组中差异均无统计学意义（均*P*>0.05）。

**表2:** 新冠疫情解控前后不同年龄段健康移植供者巨细胞病毒（CMV）和EB病毒（EBV）感染情况（％）

感染率类型	<18岁组			18～50岁组			>50岁组		
	解控前	解控后	*P*值	解控前	解控后	*P*值	解控前	解控后	*P*值
CMV既往感染率	80.79	80.17	0.877	95.28	97.02	0.070	98.52	99.32	0.281
CMV现症感染率	0.63	0.86	0.777	0.64	0.70	0.854	0.89	0.68	0.728
EBV既往感染率	87.87	77.59	0.003	95.93	97.02	0.101	96.59	97.95	0.169
EBV现症感染率	5.20	7.76	0.270	2.71	6.67	<0.001	1.85	4.10	0.019

新冠疫情解控前后EBV的IgG阳性率在<18岁组中差异有统计学意义（87.87％对77.59％，*P*＝0.003）。EBV的IgM阳性率在新冠疫情解控前后在<18岁组、18～50岁组、>50岁组中分别为5.20％对7.76％（*P*＝0.270）、2.71％对6.67％（*P*<0.001）、1.85％对4.10％（*P*＝0.019）。

5. 健康移植供者CMV和EBV激活的多因素分析：使用Logistic回归模型分析年龄、性别、新冠疫情解控是否是导致CMV和EBV再激活的风险因素。结果显示，年龄、性别、新冠疫情解控均对CMV IgM阳性率无影响。在总体人群中，年龄段［与<18岁组相比，18～50岁组（*OR*＝0.584，95％ *CI* 0.421～0.820，*P*＝0.002），>50岁组（*OR*＝0.389，95％ *CI* 0.248～0.610，*P*<0.001）］、性别（*OR*＝1.479，95％ *CI* 1.169～1.872，*P*＝0.001）、新冠疫情解控（*OR*＝2.360，95％ *CI* 1.287～3.047，*P*<0.001）均是EBV IgM阳性的独立危险因素。

进一步将人群根据性别和年龄分亚组进行多因素Logistic回归分析。根据性别分组，新冠疫情解控（男性组：*OR*＝2.256，95％ *CI* 1.604～3.173，*P*<0.001；女性组：*OR*＝2.587，95％ *CI* 1.752～3.822，*P*<0.001）和年龄［男性组：与<18岁组相比，18～50岁组（*OR*＝0.536，95％ *CI* 0.336～0.855，*P*＝0.009）、>50岁组（*OR*＝0.375，95％ *CI* 0.208～0.677，*P*＝0.001）；女性组：与<18岁组相比，18～50岁组（*OR*＝0.678，95％ *CI* 0.411～1.117，*P*＝0.127）、>50岁组（*OR*＝0.404，95％ *CI* 0.197～0.827，*P*＝0.013）］均是EBV IgM阳性的独立危险因素。

按年龄段分组，在<18岁组中新冠疫情解控和性别均不是EBV IgM阳性的危险因素；在18～50岁组中新冠疫情解控（*OR*＝2.608，95％ *CI* 1.940～3.507，*P*<0.001）和性别（*OR*＝1.499，95％ *CI* 1.138～1.975，*P*＝0.004）均是EBV IgM阳性的独立危险因素；在>50岁组中，新冠疫情解控（*OR*＝2.222，95％ *CI* 1.101～4.484，*P*＝0.026）是IgM阳性的独立危险因素，而性别对EBV IgM阳性率的影响不显著。

## 讨论

本文通过大样本队列分析了我国近10年健康人群CMV和EBV感染情况，并分析比较新冠疫情解控前后健康人群CMV和EBV感染情况差异。

本研究中8 827名健康移植供者CMV和EBV的IgG阳性率均高于90％，且随着年龄的增长感染率逐渐升高；而CMV、EBV的IgM阳性率较低。以上数据表明我国健康人群中这两种病毒的既往感染率极高，但大多数人体内的病毒处于潜伏状态，因此现症感染样本较少。这与其他文献报道的研究结果一致[12]–[13]。

不同年龄段的人群CMV IgG阳性率差异无统计学意义，但EBV IgG的阳性率在<18岁组、18～50岁组、>50岁组中分别为86.28％、96.10％、96.84％，随年龄增加而增加，且EBV现症感染率在年龄较小的组别中较高，这与既往文献[16]中EBV感染与年龄关系的研究结果一致。但需要指出，在本研究的样本中，<18岁组和>50岁组样本量明显少于18～50岁组的样本量，较少的样本量可能会对年龄分组后的数据分析产生影响，导致分析结果的差异无统计意义。除此之外，不同个体的免疫反应可能存在显著差异，抗体滴度在不同采样时间、个体免疫状况、健康史以及其他相关因素下也可能有所差异而导致误差。

新冠感染可能激活EBV感染。Navarro-Bielsa等[17]的研究指出新冠感染是疱疹病毒激活的潜在诱因；Lehner等[18]的研究提示在需要有创通气的呼吸衰竭的新冠感染患者中有78％EBV IgM检测阳性；武汉大学人民医院的Chen等[19]的研究发现在新冠检测阳性两周内EBV被重新激活；Naendrup等[20]在因新冠感染进入重症监护室的患者中进行了CMV、EBV的PCR检测，得出结论：新冠感染重症患者是EBV和CMV再激活的高危人群。本研究发现我国新冠疫情解控后，EBV的现症感染率显著升高，特别是在新冠疫情解控的3个月内，EBV现症感染率高达8.10％，这些结果提示新冠感染可能诱导EBV再激活。新冠和EBV共激活的机制目前研究仍较少，现有研究认为新冠感染导致EBV再激活的机制可能与新冠后干扰素通路活性失调、信号通路启动导致的过度炎症反应以及CD4^+^ T、CD8^+^ T和NK细胞的耗竭有关[19],[21]–[22]。Chen等[19]发现，严重的新冠合并EBV感染患者的CD8^+^ T细胞计数显著降低，并提出了由于CD8^+^ T细胞减少导致病毒重新激活的猜测。Paolucci等[21]研究发现，CD8^+^ T细胞减少与NK细胞计数、EBV DNA水平和新冠感染严重程度之间存在相关性。进一步根据年龄分层，我们的结果显示18～50岁组中新冠疫情解控后EBV感染率较高，这可能与疫情后社交活动增加、免疫状态变化等因素相关。但本研究的局限性在于我们的回顾性资料无法获取所有供者的确切新冠感染情况，因此只能以新冠疫情解控的时间点作为分析的节点。未来的研究需要结合个体新冠感染史以进一步验证新冠感染对EBV感染的影响。

综上，本研究提示近10年我国健康人群CMV和EBV既往感染率高。新冠疫情解控后健康人群中EBV现症感染风险增加，尤其在新冠疫情解控3个月内更加显著，提示新冠感染对EBV激活的影响。
